# American Dental Association and Reorganization

**Published:** 1897-07

**Authors:** 


### Editorial.

### AMERICAN DENTAL ASSOCIATION AND REORGANIZATION.

Before another issue of this journal reaches our readers the annual conventions will have assembled at Old Point Comfort, Va., and the very important work delegated to each to perform will have been, in part, accomplished.

For many reasons this annual gathering of the several organizations will be the most important held for many years, as questions affecting vitally the interests of the dental profession will be presented, and their final adjudication will require the combined thought of many minds to effect an amicable solution.

The American and Southern Dental Associations meet with the intention of grappling with a problem which must be settled now, as it will be an unwise proceeding to continue it, and be a disturbing element in national society work if left for a future period to disentangle its many complications.

The concentration and subdivision of national work, which of necessity means vital changes in present methods, cannot be ac complished without sacrifices of the attachments of many years, and a relegation of older systems to past history. The union of the American with the Southern, which has occupied much thought for many years, is but a part of the work. This at first was the central idea, but from this has developed more enlarged views which have, in degree, submerged the original question, and forced the more important one to the surface, What shall be the character of the organization or organizations to take the place of the old?

The subject has the past year been very fully discussed in the various societies throughout the country, and the delegates from all sections should by this time be fully cognizant of all the difficulties as well as of the various proposed methods of affecting reorganization that will meet all demands.

The general trend of thought seems to be towards the formation of an entirely new central body, at least new as to name if not new as to systems of work. The condition of the present organized efforts, while suitable for developing the profession, have not been found to meet the requirements of the advanced thought and practice of the present, and will most assuredly fail to satisfy the needs of the future.

The main question of a union of effort is not, from present indications, one that will meet with extended opposition. That it will occasion some friction is to be expected, but the sentiment has been continuously growing that the time has arrived when this must be accomplished, and it is the duty of all to sacrifice sentiment and long attachments to professional needs.

It is presumed that this will be the least difficulty to be overcome at Old Point Comfort. The widely diverse opinions as to methods of reorganization will doubtless be found more difficult of settlement.

The adoption of a constitution and rules for the government of any new organization is one of the most troublesome that all bodies have to meet, and it is to be hoped that the committee having this in charge will present a form of rules so clearly stated that they will avoid the necessity for long, tedious, and often times irrelevant debates.

The method usually adopted is to base an organization on the plans of similar bodies in the past, both in medicine and dentistry, with no attempt to engraft ideas more in consonance with the advanced thought of the present time and better adapted to the requirements of a more active future.

Without any desire to radically change old systems, the writer feels that to simply change names and not methods would be no gain, but rather an almost irreparable loss, as, in all probability, the opportunity for decided advance will not again occur in a generation. It, therefore, seems to him that the subject should be met not in a narrow spirit, but with an earnest desire to make the principal and governing organization worthy to represent the dental profession of America.

The suggestions that arise in the mind of the writer may not meet this demand, but, in his judgment, should form the bases of all future dental organizations. The subordinate societies may, perhaps, be arranged more effectually on present methods, but whatever may be adopted in regard to these the objective aim should be of an educational character. This is only partially accomplished by the very loose methods prevailing. These are, first, papers and, second, discussions. Very little attempt is made to regulate these, and the result is an immense amount of verbiage with a small quantity of solid pabulum. This could all readily be changed by more positive systems, requiring original work on the part of the members and limiting discussions to the subject-matter of the treatise presented. The local organizations should be strictly educational, and to attain this something more than we have at present should be adopted.

State Associations are assuming more and more legislative functions and have practically ceased to be scientific bodies. It is not possible, apparently, to change this, for their character is predetermined by the increasing tendency to law-making and law-enforcement, the end of which cannot now be foreseen. These bodies, therefore, may be left for time and circumstances to modify and perhaps destroy, for they are an anomaly in scientific thought as at present arranged.

The true scientific organizations may, therefore, be classed with the local, district, and national bodies. From the lowest to the highest there should be intimate and graded scientific relations.

To meet this demand there should be an entire change in the plans of representation. The local bodies being the source of power, the greatest care should be made to have these self-training, and as all persons require force of some kind to compel labor, this must be found either in the natural incentive to original work or in the rules governing the association. This applies with equal power to the district societies. In simple terms, the members should be trained here to exact methods, and, whether it be the simple or complex, they should be forced to give to it their undivided interest, for one clearly expressed idea, with an original flavor, is worth whole reams of platitudinous expressions. Such training is not an impossibility.

This foundation simplifies the formation of the national body. To this organization delegates should be sent, not as now, indiscriminately, but upon a basis of original work. The individual member of the local or district association who has exhibited a capacity for original work through essays presented, should be delegated to the parent body, and that for a period long enough to make him thoroughly conversant with the work. Delegates should be selected also for varying periods, so that the central body should never be left without an active working contingent. The present "permanent membership" should be quietly permitted to sink into oblivion, and in its place might be adopted an honorary, permanent membership, given only to those who have spent a good portion of their lives in original labor and have thereby added to a knowledge not possible of attainment by the majority. The number in this class would be small, but it would be an honor to those attaining it, and a prize worth the seeking. The membership of this national body, composed as it should be of the best scientific elements, would give a character to the organization similar to that possessed by leading associations now working in European countries. It would do more than this, for it would raise the standard of the dental profession to a degree that would command the respect of all cultivated persons.

The profession of dentistry to-day cannot afford to lean on the past. It must move forward or retrograde. It is, therefore, our highest duty now to let the "dead past bury its dead," and seek broader methods, that the present advanced professional education may have a field for fuller development. At no period in dentistry the world over has there been a more promising outlook. Hosts of young men are being sent out from the dental colleges trained as never before. They are, as a rule, truly professional men, and, in the near future, this should have a pronounced influence upon the intelligence of the period. It will, therefore, be a positive error if plans fail to be devised to place this growing intelligence where it will do the most effectual work.

In reorganizing, let it not be on antiquated constitutions, but aim to have these so framed that they will draw the educated, but at present isolated, elements and ingather them to the fold, and thereby strengthen the dental profession from the lowest to the highest organization. Thus arranged, the work would be worthy the ambition of any individual. Are these ideas Utopian?

